# Food sharing with friends and acquaintances: A study in preschool boys and girls

**DOI:** 10.3389/fpsyg.2023.1130632

**Published:** 2023-03-09

**Authors:** Elizabeth T. Hallers-Haalboom, Marjolijn M. Vermande, Edwin J. C. van Leeuwen, Elisabeth H. M. Sterck

**Affiliations:** ^1^Department of Clinical Child and Family Studies, Faculty of Social and Behavioural Sciences, Utrecht University, Utrecht, Netherlands; ^2^Department of Biology, Faculty of Science, Utrecht University, Utrecht, Netherlands; ^3^Department of Comparative Cultural Psychology, Max Planck Institute for Evolutionary Anthropology, Leipzig, Germany; ^4^Animal Science Department, Biomedical Primate Research Centre, Rijswijk, Netherlands

**Keywords:** sharing behavior, relationship, food preference, previous experience, sex, age

## Abstract

**Introduction:**

The current study examined whether preschoolers in a (semi-)natural situation shared more food with friends or acquaintances, and whether this was different between boys and girls, older and younger children, and for preferred and non- preferred food. In order to do so, we replicated and extended the classical work of Birch and Billman in a Dutch sample.

**Methods:**

Participants included 91 children aged between 3 to 6 years (52.7% boys, 93.4% Western European) from a middle- to upper-middle-class neighborhood in the Netherlands.

**Results:**

The results revealed that children shared more non-preferred than preferred food with others. Girls gave more non-preferred food to acquaintances than to friends, whereas boys gave more to friends than to acquaintances. No effect of relationship was found for preferred food. Older children shared more food than younger children. Compared to acquaintances, friends made more active attempts to get food. Moreover, children who were not shared with were just as likely to share food as children who were shared with.

**Discussion:**

Overall, only a small degree of agreement with the original study was found: Some significant findings could not be replicated, and some unconfirmed hypotheses of the original study were supported. The results underscore both the need for replications and studying the effect of social-contextual factors in natural settings.

## Introduction

Prosocial behavior has been broadly defined as voluntary actions intended to benefit others (Hay, 1994; Eisenberg et al., 2006) and comprises a diverse set of behaviors, such as helping, sharing, comforting, and cooperating (Dirks et al., 2018). In most societies, prosocial behavior is highly valued and an important marker of competence in children. Prosocial behavior not only contributes to the well-being of others, but also to that of the benefactors (Curry et al., 2018; Mesurado et al., 2019; Hui et al., 2020).

To foster better understanding of other-oriented behaviors, Dunfield (2014) proposed that the broad class of prosocial behavior should be divided into more specific behaviors that each address a unique negative state in the other (e.g., emotional distress in the case of comforting, instrumental need in the case of helping) and require distinctive social cognitive demands to alleviate this need. As a result, it becomes easier to understand why different prosocial behaviors are not or only minimally correlated with each other and follow separate developmental trajectories (Dunfield, 2014; Padilla-Walker and Carlo, 2014; Song, 2019). According to this taxonomy, the prosocial act of sharing (i.e., giving up a (limited) resource; Hay, 1979) is elicited by another’s unmet material desire. To effectively alleviate this negative state requires the ability to recognize unequal distribution of resources, the motivation to see equality restored, and the ability to overcome an egocentric desire to monopolize resources (Dunfield, 2014).

The priors of sharing, such as the ability to understand another’s unmet material desire and the unequal distribution of sharing, appear early in life. During the second year of life, children begin to be able to overcome their own material desire to have resources, as shown by increases in passive (tolerated theft) and active sharing (either spontaneous or instigated by the interaction partner; Eisenberg et al., 2006; Dunfield, 2014; Song, 2019). However, sharing behavior appears to be rather complex and preschool children do not share in every situation to the same extent (Paulus and Moore, 2014; Martin and Olson, 2015). A wide array of social-contextual factors have been proposed to affect sharing behavior, but findings are not always consistent and many questions about the nature of sharing and the motivations that lead to decisions of young children to share are still unanswered (Dunfield, 2014; Martin and Olson, 2015; Dirks et al., 2018).

Due to the limited cognitive abilities of young children, researchers have often used parent and teacher reports to assess sharing and the mechanisms influencing it (Eisenberg and Fabes, 1998). Questionnaire data have the disadvantage that they do not always reflect actual prosocial behavior (e.g., Kogut, 2012). More recently, specific standardized tasks have been applied. In these tasks, adult experimenters often stimulate/scaffold young children’s sharing behavior (e.g., Dunfield et al., 2011) and/or use puppets (e.g., Chernyak and Sobel, 2016; Ulber et al., 2017) or imaginary others (e.g., Blake and Rand, 2010; Liu et al., 2016) to study sharing. Although these studies have contributed significantly to our understanding of children’s sharing, experimental studies do not always resemble children’s natural social context. As children enter school or daycare, their social experiences with (same-age) peers become increasingly important (Eisenberg et al., 2006). Compared to the experimental studies above, interaction with real peers may be more cognitively challenging (Song, 2019) and more dependent on the behavior of the peer (Birch and Billman, 1986). Yet, a recent meta-analysis showed that most of the studies on sharing in infancy through adolescence used experimental tasks rather than (semi-)natural observations in schools or daycare (Song, 2019).

Of the resources used to study sharing by young children, food occupies a special place. First of all, food sharing is one of the most common cross-cultural manifestations of sharing (Bird et al., 2018) and allows for cross-cultural comparisons (Rao and Stewart, 1999). Secondly, studying food sharing enables us to compare human and nonhuman primates to unravel the evolutionary roots of sharing behavior (Hare et al., 2007; Jaeggi et al., 2010). Thirdly, sharing of food tends to be more definitive than sharing of objects (e.g., toys or stickers), as there is a high probability that the recipient will eat the food immediately. Finally, children appear to value food (particularly candy) more than other types of resources (Murnighan and Saxon, 1998). Correspondingly, a recent meta-analysis found that during preschool age sharing increased when the resources were toys, but no increase was found when sharing food (Song, 2019).

One of the few studies that examined young children’s food sharing at school and multiple social-contextual factors influencing it, was conducted by Birch and Billman (1986). Owing to the encompassing nature, their study has rather uniquely transcended scholarly disciplines, as indicated by the diversity of research fields citing the work (ranging from developmental psychology to behavioral biology). Using a semi-naturalistic situation, they observed 57 pairs of same-sex American preschoolers during food sharing sessions with friends and acquaintances at school, where the potential sharer had 10 pieces of preferred and 10 pieces of non-preferred food and the friend/acquaintance only one piece of each. In that way, they were able to study how age and sex, the relational context (friends vs. acquaintances), the value of the resources (high vs. low), and social experience (previous experience as a recipient) influence young children’s sharing, aspects that still need controlled investigations today (Dirks et al., 2018).

One of the main conclusions drawn by Birch and Billman (1986) was that girls shared more food with friends than with acquaintances, while boys did not share differently across friends or acquaintances. According to the authors, this fits with the idea that boys and girls differ in their social experiences and friendship patterns; girls distinguish more clearly between friends and others, whereas boys form larger, more fluid, extensive groups. Further, most sharing was triggered by soliciting, with friends more actively soliciting for food than acquaintances. The behavior of the potential recipient thus seems to play an important role in determining whether or not sharing occurs, and (elicited) sharing might be particularly a component of friendship (i.e., one of the things expected of a friend). Lastly, although there was no effect of simply having previous experience as a recipient or not, the authors concluded that preschoolers’ sharing behavior appeared to be influenced by the *quality* of their previous experience as a recipient. Almost all children who were shared with subsequently shared themselves, whereas only half of the children who were not shared with did so, supporting the idea of modeling in the socialization of sharing among young children (Birch and Billman, 1986). Surprisingly, no effects of age and food preference were found.

Despite its many strengths, Birch and Billman’s (1986) study has several shortcomings. One limitation is that their conclusion that girls share more with friends than with acquaintances, whereas boys do not, was based on multivariate results on food in general that were only supported by univariate results on *non*-preferred food (and not by results on preferred food). Moreover, the finding that girls gave more disliked food to friends than acquaintances whereas boys did not, does not fit with their interpretation stated above in terms of differences in boys’ and girls’ social experiences and friendship patterns and is therefore rather counterintuitive. In addition, in the Discussion of their paper, the authors suggest that the lack of an effect of food preferences on sharing (i.e., children did not share more readily non-preferred than preferred food as predicted) could be explained by the observation that children (particularly boys) often “dumped” the non-preferred food despite the recipient’s protest and sometimes giving it back. Birch and Billman therefore argued that dumping is not actually prosocial behavior, as it does not involve either self-sacrifice by the sharer or benefit to the other. However, this was progressive insight; they did not examine this possibility.

### Current study

The goal of the current study was to conduct a direct replication (LeBel et al., 2018) of Birch and Billman’s (1986) classical study on food sharing in preschool children, while dealing with the above-mentioned shortcomings. Because of both the remarkable results and lack of studies in this area, we were especially interested in the absence of an effect of food preference (i.e., children did not share more non-preferred food compared to preferred food) and the aforementioned interaction between relationship quality and sex (i.e., girls gave more disliked food to friends than acquaintances, whereas boys did not). Our main hypotheses were based on Birch and Billman’s (1986) results (see also Table 1) and recent literature when available.

**Table 1:** Overview and comparison of results by Birch and Billman (1986) and the current study.

	Birch and Billman (1986)	Current study
**Quantity of sharing (frequency and amount)**		
Preferred (favored) food	Relationship: Children shared more preferred food with friends than with acquaintances.	Relationship: No effect
	Sex: No effect	Sex: No effect
	Age: No effect	Age: Older children shared more preferred food with others than younger children did.
	Previous experience: No effect	Previous experience: No effect
Non-preferred (disliked) food	Relationship*Sex: Girls shared more non-preferred food with friends than with acquaintances. For boys, patterns of sharing did not differ between friends and acquaintances.	Relationship*Sex: Girls shared more non-preferred food with acquaintances than with friends, whereas boys gave more non-preferred food to friends than to acquaintances.
	Age: No effect	Age: Older children more often shared non-preferred food with others than did younger children.
	Previous experience: No effect	Previous experience: No effect
Food preference	No effect	Children shared more non-preferred food than preferred food with others.
**Type of sharing**		
Spontaneous	Relationship: No effect	Relationship*Sex: Girls shared more (non-preferred) food spontaneously with acquaintances than with friends, but for boys patterns of sharing did not differ between friends and acquaintances.
	Sex: No effect	
	Age: No effect	Age: No effect
	Previous experience: No effect	Previous experience: No effect
Elicited	Relationship: More food was shared more frequently with friends than with acquaintances.	Relationship: Elicited sharing was more frequent among friends than acquaintances.
	Sex: No effect	Sex: No effect
	Age: No effect	Age: No effect
	Previous experience: No effect	Previous experience: No effect
Passive	Relationship: No effect	Relationship: Passive sharing was more frequent among friends than acquaintances.
	Sex: No effect	Sex: No effect
	Age: No effect	Age: Passive sharing was more frequent among older children than younger children.
	Previous experience: No effect	Previous experience: Passive sharing was more frequent among children without previous experience as a recipient.
**Previous experience**		
Type of experience	A successful experience as a recipient facilitated subsequent sharing, whereas unsuccessful experiences did not.	Children who were not shared with were just as likely to share food as children who were shared with.

First, Birch and Billman (1986) rather unexpectedly found no effect of food preference on sharing. The effect of resource value has received little attention in previous research, as many experiments only used one type of tokens (often of uncertain or potentially little value to children; Shaw and Olson, 2013). To date, a few recent studies showed that even young children take resource value into account when deciding how to minimize inequality in outcomes between others (e.g., children donating more of their least favorite sticker than their favorite sticker; Blake and Rand, 2010; Shaw and Olson, 2013). Moreover, a recent meta-analysis showed that individuals are less generous in a Dictator Game when the stakes are higher (Larney et al., 2019). A possible explanation for this finding is that sharing less desirable resources requires less self-sacrifice compared to the sharing of more desirable ones. Therefore, we still expected preschoolers to give more disliked food than favored food.

Second, Birch and Billman (1986) found an interaction effect of relationship quality and sex on food sharing. As explained above, the finding that girls gave more disliked food to friends than acquaintances, whereas boys did not, does not fit with their own description of differences in boys’ and girls’ experiences and friendship patterns. Given that more recent literature underscores sex differences in peer relations (i.e., girls’ relationships seem to be characterized by prosocial behavior to a greater degree than boys and girls tend to distinguish more clearly between friends and other peers than boys; Schneider et al., 2005; Rose and Rudolph, 2006; Martin et al., 2018), we therefore expected that girls would give *less* disliked food to friends than acquaintances. Following this train of thought, it could also be expected that girls would give more favored food to friends than acquaintances, but Birch and Billman found no such effect. Instead, their results showed that both boys and girls shared more preferred food with friends than with acquaintances. Recent research found additional evidence that children tend to share more objects (mostly nonfood) with friends than with either non-friends, disliked peers, strangers, or out-group members (e.g., Fehr et al., 2008; Moore, 2009; Paulus, 2016; Sparks et al., 2017; Lenz and Paulus, 2021). A recent meta-analysis, however, did not find support that sharing is dependent on relationship type (friend vs. stranger) from the preschool years to childhood, although this effect may be due to the lack of studies (Song, 2019). So, we tentatively expected that children shared more (preferred) food with friends than with acquaintances.

Third, the majority of sharing in Birch and Billman’s (1986) study was the result of active elicitation on the part of the recipients. Even in the case of the glaring inequality that was created, spontaneous sharing was very rare. Birch and Billman concluded that recipients do not simply wait for spontaneous sharing to occur, but rather were verbally and physically active in eliciting it. Within friendship dyads, recipients were particularly vigorous elicitors. This fits with other research indicating that behaving prosocially is an important component of friendship, and that children expect their friends to help and support each other (Furman and Bierman, 1983; Dirks et al., 2018). Based on these findings, we also expected friends to be more active elicitors of sharing than acquaintances.

Fourth, Birch and Billman (1986) found that the *quality* of previous experiences as a recipient influenced subsequent sharing behavior. Recent studies also found young children’s sharing behavior to be affected by others’ generosity or stinginess, but most of these studies focused on direct reciprocity (i.e., exchange of acts between the same two individuals; e.g., House et al., 2013; Warneken and Tomasello, 2013; Messer et al., 2017; Vaish et al., 2018; Wörle et al., 2020), whereas in Birch and Billman’s study children were not re-paired with the same child who shared with them before. By observing subsequent sharing with another child, Birch and Billman focused on indirect “upstream” reciprocity (i.e., paying forward an act not to the person from whom it had been received, but to a different person instead). To the best of our knowledge, only a few other studies found support for upstream reciprocity among preschoolers (Leimgruber et al., 2014; Beeler-Duden and Vaish, 2020; Wörle et al., 2020), but these studies used experiments rather than (semi-)natural observations with peers. Therefore, we tentatively expected positive previous experience as a receiver to stimulate sharing.

Fifth, although Birch and Billman (1986) did not find an effect of age or sex on sharing, recent studies indicated that sharing behavior increases with age (Fehr et al., 2008; Blake and Rand, 2010; Malti et al., 2016). Moreover, a recent meta-analysis reported that older preschoolers (mean 68.79 months) showed more sharing than early preschoolers (mean 54.9 months; Song, 2019). Although sex was not included in this meta-analysis, other studies suggested higher levels of prosocial behavior among girls than boys (Eisenberg and Fabes, 1998; Blake and Rand, 2010). However, the effects were small for sharing and in case the target was another child (Eisenberg and Fabes, 1998). Among adults, sex differences in generosity are however well-established (i.e., women tend to share more than men; Engel, 2011; Rand et al., 2016; Brañas-Garza et al., 2018). Therefore, we tentatively expected an effect of age (i.e., more sharing among older children) and sex (i.e., more sharing among girls).

## Method

### Participants

Birch and Billman’s (1986) sample consisted of 57 3-5-year-old children from five classrooms at the University of Illinois Child Development Laboratory. To obtain a similar sample, we recruited participants from five Kindergarten classes with 4–6 year-old children of an elementary school and one preschool class offering early education to 2–4 year-olds in the Netherlands. Both schools were situated at the same location in a middle- to upper-middle-class neighborhood. After obtaining consent from the schools and classroom teachers, all parents received a brochure with information about the procedures and data storage and were asked to return a signed consent form. Children who only recently entered school and did not yet have formed relationships with their classmates could not participate. Consent was obtained for 61.5% (*N* = 96) of the eligible children. Children with food allergies (*n* = 1) and children with missing data due to technical failure or unexpected absence from school (*n* = 4) were excluded, resulting in a final sample of 91 children from six classes (age range 2.8 to 6.5 years; *M*_age_ = 4.88, *SD* = 0.96; 52.7% boys). Most of the children were Dutch (90.1%), the other children originated from Western (3.3%) or non-Western (6.6%) countries other than the Netherlands.

### Procedure

To replicate the procedure as precisely as possible, one of the authors of the original publication (dr. L. L. Birch) was contacted. Following the original study and Billman’s (1984) thesis, all children completed assessments to obtain information about their sociometric choices and food preferences. Based on this information, the children were then paired with a friend and with an acquaintance on two different occasions, separated by a period of about 2 weeks. The order in which children were coupled with a friend or acquaintance was counterbalanced. The observations consisted of a snack moment, during which the children had the opportunity to share most- and least-preferred food. The potential sharer received large quantities of the food relative to the recipient. All sessions were filmed and were led by pairs of trained (under)graduate students. For each session, the pair of children was taken from their classroom to a separate room where they could not be disturbed by others. Ethical approval for this study was provided by the Faculty Ethics Review Board (FERB) of the Faculty of Social and Behavioural Sciences of Utrecht University, the Netherlands.

According to LeBel et al. (2018) Replication Continuum, our study can be considered a direct and “very close” replication of the original study with only differences in contextual variables beyond a researcher’s control (e.g., history, culture, language) and procedural details.

### Measurements

#### Sociometric tests

Photographs of the children’s heads and torsos were made. These photographs served as the stimuli in the Paired-Comparisons Sociometric Test (PCST) and the Peer Preference Assessment (PPA). Two tests were used, as convergence of the information would provide a more valid assessment of the children’s friends and acquaintances (Birch and Billman, 1986). The two tests were administered 1 week apart to ensure that the relationships between children were stable. The PCST was administered before the PPA.

For both sociometric tests, children judged same-sex peers within their classroom by their likeability. In the Birch and Billman (1986) study, children and their peers differed at most 8 months in age. In the present study, this restriction regarding the age of the children was not feasible, because in the Netherlands the two lowest grades of elementary school (i.e., Kindergarten) are usually combined into composite classes, which was also the case in the present sample. As a result, children may form relationships with older or younger classmates and restricting the age range would lower the ecological validity. The number of peers to evaluate ranged from 3 to 10 children.

##### Paired-comparisons sociometric test

The children were presented with all possible combinations of pairs of photographs of their same-sex classmates. The number of possible pairs ranged from 3 to 45. The order in which the pairs were presented and the position of the photographs (i.e., on the left or right side) were randomly determined. For each pair of photographs, the child was asked to choose the person they would like to play with the most. Each time a classmate was chosen from a pair, that classmate was given a point. At the end, all choices by the child were summed to obtain a rank order for the (same-sex) peers.

##### Peer preference assessment

The children were asked to place each photograph (in random order) into one category: “like to play with,” “do not like to play with,” or “just okay.” Pictures of three gender-neutral smiley faces, varying in expression (happy, sad, and neutral), were used to represent the categories. After categorizing all photographs, the child then had to rank the photographs within each category (i.e., through repeated nominations of the best liked person left in that category), resulting in a complete rank order.

##### Dyad selection

For each child, the designated friend was determined by selecting the classmate who was ranked first or second on the PCST and who was categorized in the “like to play with” category on the PPA. Acquaintances were selected by using the following criteria: (i) the acquaintance was never the least-liked classmate on either measure (to eliminate children who were disliked), (ii) the acquaintance was never the first or second-best-liked classmate on either of the measures, (iii) the acquaintance was preferably rated in the “neutral” category on the PPA. In case several options were available, the selection for the designated acquaintance was random. In the eight ambiguous cases that did not fit all three criteria, the child’s teacher was consulted to make decisions.

#### Food preference assessment

The procedure of the Food preference assessment (FPA; Birch et al., 1980) was similar to the PPA described above. Pictures of three gender-neutral smiley faces were used, varying in expression (happy, sad, and neutral). Similar to Birch and Billman (1986), children were presented with seven samples of foods in small, clear-plastic cups: M&M’s, mini-marshmallows, Pepperidge Farm Goldfish Crackers, bite-sized pieces of cheese, (uncooked) carrot slices, bite-sized pieces of raw broccoli, and bite size pieces of radish. The child was asked to taste each food and place the cup in front of the face that matched with their affective response to the food. After categorizing all the foods, the experimenter focused the child’s attention to the set of foods that were placed in the “like” category and asked the child to select the very best-tasting food. Then, the child was asked to choose the best-tasting food from the remaining food in that category. The same procedure was applied to the “neutral” and “dislike” categories, resulting in a complete rank order of the food.

#### Snack-time sharing and coding

The snack-time sessions started approximately 1 week following the second sociometric test and were planned right before the regularly scheduled snack moment at the school. In the Birch and Billman (1986) study, children each received a small brown bag with their name. The bag of the potential sharer contained 10 pieces of his/her preferred and non-preferred food and the bag of the potential recipient contained one piece of each of the same foods. Based on a pilot with 12 children, a small adjustment in this procedure had to be made. The main reason for this was that the children in our study were not used to getting a snack in a bag marked with their name, as schools in the Netherlands generally do not provide snacks to the children (but children bring their own snack of choice from home). Because we could not create a situation that exactly resembled that of the Birch and Billman study, we instead used one bag with food for each pair of children and the experimenter then distributed the food as in the original study. Prior to the session, the bags were prepared with the target child’s most preferred and least preferred foods. After the children were seated at the table, the experimenter gave each of the children their name-labeled paper plate and showed them the bag of food. While the experimenter put the food on the children’s plates, she asked the children to wait until she was done. All the food was first placed on the plate of the target child, after which the experimenter took one piece of the preferred and non-preferred food and put it on the plate of the non-target child. As a result, the target child received 10 pieces of each food, and the non-target child received only one piece of each of the same foods. The experimenter then said she suddenly had to go, but that the children could eat while she was gone. The children were videotaped by a second experimenter. Sessions were limited to 5 minutes, after which the experimenter returned and escorted the children back to their classroom.

Each child was observed twice as a potential sharer, once with a friend and once with an acquaintance. The order of these observations was counterbalanced, resulting in a group of 43 children first seen with a friend and 48 first seen with an acquaintance. Further, scheduling was arranged in such a way that two groups were formed: one group of children who had no previous experience as a potential recipient (*n* = 41) and one group who had already been a recipient in the snack session once (*n* = 50).

Coding procedures were based on Birch and Billman (1986) and Billman (1984). Both *number of sharing incidents* (i.e., occurrences during which the target child shared preferred and/or non-preferred food with the recipient; frequency of sharing) and the *number of pieces of preferred and non-preferred food* shared with the recipient were counted. When a child took food back from the recipient after sharing, this was subtracted from the total number of food shared. Further, three types of sharing modes were coded: spontaneous, elicited, and passive. *Spontaneous sharing* was coded when the target child took the initiative for sharing, without any prior verbal or physical behaviors on the part of the recipient to get food. *Elicited sharing* was coded when the target child shared food in response to verbal (e.g., asking, demanding) or physical actions (e.g., extending hands, pointing) of the recipient to get food (i.e., the recipient’s behavior was instrumental in initiating sharing). *Passive sharing* was coded when the target child allowed the recipient to take food of their plate (i.e., the initiative for sharing was taken by the recipient). For each sharing mode, the *number of sharing incidents* and *number of pieces of food* were counted. With respect to sharing incidents, a distinction was made between successful and unsuccessful elicitations and attempts to take or share food. No distinction between elicitations for preferred and non-preferred food was made, because according to Birch and Billman (1986; p. 392) it was not always possible to specify whether the elicitations referred to preferred or non-preferred food.

Three trained coders rated the videotapes. To guarantee independence among ratings, no coder rated a target child twice. Coder reliabilities were computed on 22% of the videotapes (*n* = 40). The mean intraclass correlation coefficient (absolute agreement) for incidents sharing preferred food was 0.96 (range 0.94–0.98), for incidents sharing non-preferred food was 0.96 (range 0.95–0.98), for pieces of preferred food was 0.99 (range 0.99–0.99), for pieces of non-preferred food was 0.99 (range 0.99–1.00), for spontaneous sharing was 0.96 (range 0.94–0.99), for elicited sharing was 0.92 (range 0.86–0.97), and for passive sharing was 0.93 (range 0.80–0.99). During the coding process, 20% of the videotapes (*n* = 37) were coded twice by separate coders to prevent coder drift. Further, all coding forms were double checked to ensure that no counting errors were made.

### Data-analyses

We followed the same analytic strategy as in the original study. Following Birch and Billman (1986), the sample was divided into a younger (≤50 months, *n* = 17) and an older group (>50 months, *n* = 74). Analyses of children’s food sharing with friends and acquaintances were conducted using GLM Repeated Measures MANOVA, as was done in the original study. The analysis regarding the *quantity of sharing* (i.e., frequency of sharing and amount of food shared) included the following dependent variables: number of incidents sharing preferred food, number of pieces of preferred food shared, number of incidents sharing non-preferred food, number of pieces of non-preferred food shared. For the analyses regarding the *type of sharing* (i.e., spontaneous, elicited, passive sharing), separate analyses were conducted. The dependent variables in these analyses were the number of sharing incidents (i.e., both successful and unsuccessful) and number of pieces of food shared within the sharing mode of interest. For all analyses, main effects and two-way interactions between the within-subjects factor (relationship) and between-subjects variables (age, sex, having previous experience as a recipient, friend-acquaintance/acquaintance-friend order) were examined. Birch and Billman did not report higher order interaction effects. For the interpretation of results, we first evaluated (significant) two-way interactions and then assessed any additional main effects. Moreover, in case of significant multivariate effects, the univariate analyses were inspected for further clarification.^1^

## Results

### Quantity of sharing (frequency and amount)

Table 2 shows the mean number of sharing incidents and mean number of pieces of preferred and non-preferred food shared with friends and acquaintances, separately for boys and girls. The two measures of preferred food were strongly positively associated, *r* = 0.81, *p* < 0.001. The same pattern was found for the measures of non-preferred food, *r* = 0.57, *p* < 0.001. When looking at correlations between preferred and non-preferred food, medium positive associations for sharing incidents, *r* = 0.30, *p* < 0.01, and pieces of food, *r* = 0.22, *p* < 0.05, were found. Similar patterns of correlations were found in the original study (see Birch and Billman (1986), p. 391).

**Table 2:** Means and standard deviations for sharing incidents and number of pieces of preferred and non-preferred food shared with friends and acquaintances, separately for boys and girls.

	Boy (*n* = 48)	Girl (*n* = 43)	Total (*n* = 91)		*F*(1,86)	*η_p_^2^*
*M (SD)*		*M (SD)*	*M (SD)*	Range		
Incidents P					1.11	0.013
Friend	1.56 (1.47)	1.70 (1.37)	1.63 (1.42)	0–6		
Acquaintance	1.17 (1.24)	1.47 (1.49)	1.31 (1.36)	0–5		
Incidents NP					0.79	0.009
Friend	1.96 (1.76)	1.70 (1.73)	1.84 (1.74)	0–8		
Acquaintance	1.42 (1.71)	1.77 (1.82)	1.58 (1.76)	0–7		
Pieces P					0.17	0.002
Friend	2.93 (2.51)	1.93 (1.64)	2.46 (2.19)	0–8.5^1^		
Acquaintance	2.04 (2.35)	2.27 (2.39)	2.15 (2.36)	0–10		
Pieces NP					0.003	<0.001
Friend	5.38 (4.13)^a^	2.38 (2.82)^a^	3.96 (3.86)	0–10		
Acquaintance	3.52 (3.98)^b^	3.95 (3.82)^b^	3.73 (3.89)	0–10		

P, preferred food; NP, non-preferred food. *F*-value represents the univariate results for the effect of relationship (friend vs. acquaintance). Different superscripts indicate significant differences within columns.

1 If children broke a piece of food in half, only half a piece was scored.

Table 1 gives an overview of Birch and Billman’s (1986) results. They concluded that no overall effect of food preference was present. The findings of the current study, however, indicated that children shared more pieces of non-preferred food than preferred food with both friends, *t*(90) = −3.86, *p* < 0.001, *d* = 0.48, and acquaintances, *t*(90) = −3.61, *p* = 0.001, *d* = 0.49 (both small-medium effects). No differences were found regarding the number of incidents (*p*-values ranged from 0.186 to 0.306). Interestingly, after taking dumping into account, we no longer found any differences in the amount of preferred and non-preferred food shared with others (*p*-values ranged from 0.232 to 0.615). See also Supplementary information.

**Figure 1:**
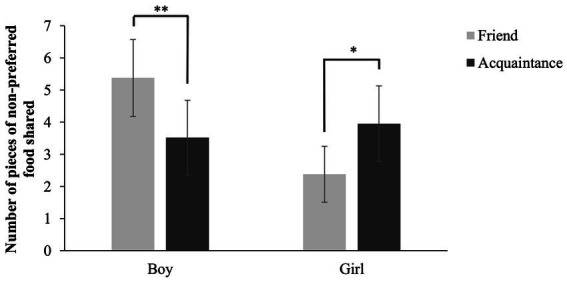
Interaction between number of pieces of non-preferred food shared and sex. Error bars represent 95% confidence intervals. *p<0.05, **p<0.01.

Similar to Birch and Billman (1986), we found a significant multivariate interaction between relationship and sex, *Pillai’s F*(4,83) = 4.63, *p* = 0.002, *η_p_^2^* = 0.182 (large effect). The univariate analyses, however, revealed only a significant interaction when comparing the number of pieces of non-preferred food shared by boys and girls, *F*(1,86) = 13.08, *p* = 0.001, *η_p_^2^* = 0.132 (medium-large effect). Follow-up paired *t*-tests showed that boys shared more pieces of non-preferred food with friends than with acquaintances, *t*(47) = 2.97, *p* = 0.005, *d* = 0.46, whereas girls gave more pieces of non-preferred food to acquaintances than to friends, *t*(42) = −2.43, *p* = 0.020, *d* = 0.47 (Figure 1). This result is in the opposite direction as the original study. Although in the original study also an interaction between relationship and sex was found for the number of incidents sharing non-preferred food, this was not the case in our study (*p* = 0.208).

Birch and Billman (1986) further found a significant univariate main effect of relationship for both measures of preferred food variables (i.e., with respect to preferred food, children shared more and more often with friends than with acquaintances), but we did not find such effects in our study (*p*-values ranged from 0.294 to 0.678).

No other effects were significant in Birch and Billman’s (1986) study. However, as hypothesized by Birch and Billman, in the current study additional significant main effects were found for age: older children engaged in sharing non-preferred food more often than younger children, *F*(1,86) = 4.97, *p* = 0.028, *η_p_^2^* = 0.055 (small-medium effect), and also shared more pieces of preferred food than younger children, *F*(1,86) = 4.75, *p* = 0.032, *η_p_^2^* = 0.052 (small-medium effect). None of the other effects were significant (*p*-values ranged from.067 to.976).

### Type of sharing

For each sharing mode (spontaneous, elicited, passive), Table 3 shows the mean number of sharing incidents and mean number of pieces of food shared with friends and acquaintances separately for boys and girls. Whereas Birch and Billman (1986, p. 392),^2^ reported relatively low frequencies of spontaneous sharing, children in our study clearly shared more food spontaneously (Table 2). The two measures (i.e., number of sharing incidents, number of pieces of food) of spontaneous sharing were strongly positively associated, *r* = 0.65, *p* < 0.001. The same pattern was found for elicited sharing, *r* = 0.44, *p* < 0.001, and passive sharing, *r* = 0.73, *p* < 0.001. Notably, the correlations for our measures were somewhat lower than in the original study.

**Table 3:** Means and standard deviations for spontaneous, elicited, and passive sharing with friends and acquaintances, separately for boys and girls.

	Boy (*n* = 48)	Girl (*n* = 43)	Total (*n* = 91)		*F*(1,86)	*η_p_^2^*
*M (SD)*		*M (SD)*	*M (SD)*	Range		
**Spontaneous sharing**						
Incidents					0.35	0.004
Friend	1.33 (1.45)	1.58 (1.67)	1.45 (1.55)	0–6		
Acquaintance	1.29 (1.61)	1.84 (1.94)	1.55 (1.78)	0–7		
Pieces					2.13	0.024
Friend	3.60 (4.01)	1.64 (2.10)^b^	2.68 (3.38)	0–10		
Acquaintance	2.81 (4.00)	3.43 (3.65)^a^	3.10 (3.83)	0–12		
**Elicited sharing**						
Incidents					4.42^*^	0.049
Friend	1.15 (1.86)	1.35 (1.41)	1.24 (1.66)^a^	0–8		
Acquaintance	0.88 (1.36)	1.00 (1.27)	0.93 (1.31)^b^	0–7		
Pieces					0.02	<0.001
Friend	0.97 (1.78)	0.86 (1.79)	0.92 (1.78)	0–10		
Acquaintance	0.85 (2.31)	1.01 (2.23)	0.93 (2.26)	0–11		
**Passive sharing**						
Incidents					5.04^*^	0.055
Friend	2.15 (2.10)	1.88 (1.95)	2.02 (2.03)^a^	0–10		
Acquaintance	1.19 (1.68)	1.23 (1.54)	1.21 (1.61)^b^	0–6		
Pieces					2.56	0.029
Friend	3.50 (4.01)	1.79 (2.42)	2.69 (3.44)	0–13		
Acquaintance	1.90 (2.78)	1.78 (2.85)	1.84 (2.80)	0–10		

*F*-value represents the univariate results for the effect of relationship (friend vs. acquaintance). Different superscripts indicate significant differences within columns.

* *p* < 0.05.

#### Spontaneous sharing

**Figure 2:**
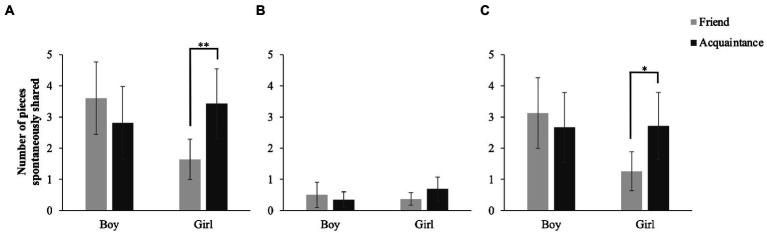
Interaction between number of pieces of spontaneously food shared and sex. **(A)** Total number of pieces of food shared spontaneously. **(B)** Number of pieces of preferred food shared spontaneously. **(C)** Number of pieces of non-preferred food shared spontaneously. Error bars represent 95% confidence intervals. *p<0.05, **p<0.01.

The initial study did not find any (multivariate) significant interaction effects or main effects for spontaneous sharing, but in the current study a significant multivariate interaction between relationship and sex was found, *Pillai’s F*(2,85) = 6.07, *p* = 0.003, *η_p_^2^* = 0.125 (medium-large effect). Within-subjects contrasts revealed a significant interaction when comparing the number of pieces shared spontaneously by boys and girls, *F*(1,86) = 7.99, *p* = 0.006, *η_p_^2^* = 0.085 (medium effect). Girls spontaneously shared more food with acquaintances than with friends, *t*(42) = −3.16, *p* = 0.003, *d* = 0.60 (Figure 2A). For boys, patterns of spontaneous sharing did not differ between friends and acquaintances, *t*(47) = 1.26, *p* = 0.205, *d* = 0.20. No other effects were significant (*p*-values ranged from 0.087 to 0.988).

Birch and Billman (1986) did not make a distinction between preferred and non-preferred food for the variables concerning spontaneous sharing. However, spontaneous sharing might be related to the target child’s preference for food. To increase our understanding of the interaction between relationship and sex for the number of pieces that were shared spontaneously, we reran our analyses separately for the amount of preferred and non-preferred food shared spontaneously. The results revealed that the relationship*sex interaction only remained present when considering the number of pieces of non-preferred food, *F*(1,86) = 5.45, *p* = 0.022, *η_p_^2^* = 0.060 (medium effect), indicating that the girls’ greater amount of food shared spontaneously with acquaintances than friends was attributable to the sharing of non-preferred food (Figure 2).

#### Elicited sharing

In line with the findings of Birch and Billman (1986), a significant multivariate main effect of relationship was found for elicited sharing, *Pillai’s F*(2,85) = 4.07, *p* = 0.021, *η_p_^2^* = 0.087 (medium effect). Contrasts revealed that elicited sharing incidents occurred more often when sharing with friends compared to acquaintances, *F*(1,86) = 4.42, *p* = 0.038, *η_p_^2^* = 0.049 (small effect). Unlike the original study, this was not found for the number of pieces shared (*p* = 0.897). No other effects were significant (*p*-values ranged from 0.285 to 0.983).

#### Passive sharing

The original study did not find (multivariate) significant interaction or main effects for passive sharing. We also did not find any significant interactions (*p*-values ranged from 0.080 to 0.976), but our results showed significant multivariate main effects of age, *Pillai’s F*(2,85) = 4.23, *p* = 0.018, *η_p_^2^* = 0.091 (medium effect), and previous experience, *Pillai’s F*(2,85) = 3.18, *p* = 0.047, *η_p_^2^* = 0.070 (medium effect). Inspection of the between-subjects tests revealed only an effect of age, *F*(1,86) = 8.35, *p* = 0.005, *η_p_^2^* = 0.088 (medium effect), and previous experience, *F*(1,86) = 5.75, *p* = 0.019, *η_p_^2^* = 0.063 (medium effect), on the number of passive sharing incidents (i.e., higher among older children and children with no previous experience). Moreover, a significant univariate main effect of relationship was found for passive sharing incidents, *F*(1,86) = 5.04, *p* = 0.027, *η_p_^2^* = 0.055 (small-medium effect). Within-subjects contrasts showed that passive sharing was more frequent among friends than acquaintances. No other effects were significant (*p*-values ranged from 0.055 to 0.780).

#### Success rate of attempts

Our results showed that the total number of elicited and passive sharing incidents were more frequent with friends than with acquaintances. For all these sharing incidents, the success of the attempts to get food was coded (see Table 4). In the original study, the greater proportion of total sharing incidents with friends appeared to be attributable to a higher rate of attempted initiations made by friends, while the success rates for friends (38%) and acquaintances (30%) did not differ significantly. In line with these findings, our results showed that the success rates of the elicited and passive sharing incidents with friends (63.5%) and acquaintances (65.9%) were also largely similar, but substantially higher compared to those reported by Birch and Billman (1986, p. 392).

**Table 4:** Mean number of successful and unsuccessful attempts to acquire food.

	Successful attempts *M* (*SD*)	Unsuccessful attempts *M* (*SD*)
**Elicited sharing**		
Friend	0.53 (0.96)	0.71 (1.25)
Acquaintance	0.42 (0.75)	0.52 (1.06)
**Passive sharing**		
Friend	1.54 (1.77)	0.48 (0.98)
Acquaintance	0.99 (1.44)	0.23 (0.56)

### Type of previous experience

The sharing behavior of the group of children with previous experience as a recipient (*n* = 50) was examined more closely following the rationale explained in the Introduction that not simply having experience as a recipient (see above), but also the *quality* of this experience might be relevant in determining subsequent sharing behavior. For one child, the type of previous experience could not be determined due to technical issues with the videotape.

Birch and Billman (1986) reported that the subgroup of children who had previous experience as recipients (*n* = 27) was nearly equally divided with respect to whether they had been shared with (*n* = 14) or not (*n* = 13). In the present study, the group of children with previous experience as a recipient (*n* = 49) appeared to be *un*equally divided with respect to whether they had been shared with (*n* = 36) or not (*n* = 13). In contrast to Birch and Billman, we found no association between the nature of their previous experience (i.e., been shared with or not) and children’s subsequent sharing behavior at T1 in our study, χ^2^(1) = 0.59, *p* = 0.442. Of the children who were shared with, the majority of them shared subsequently (*n* = 31). Only five children in this subgroup did not share with their peer. For the children who were not shared with, however, a similar pattern was found. Most of the children in this group shared subsequently (*n* = 10), whereas three children did not. A binomial test (with *mu* set to 0.5 to test against chance level) indicated that the proportion of children who subsequentially shared (86%) after a positive experience (i.e., been shared with) was in the expected direction (*p* < 0.001, 1-sided), but that the proportion of children who subsequently shared (77%) after a negative experience (i.e., not been shared with) was also higher than expected (*p* = 0.090, 2-sided).

## Discussion

The main objective of the current study was to replicate and extend Birch and Billman’s (1986) semi-natural study of food sharing in young children. We were especially interested in the effect of food preference and the interaction between relationship quality and sex on sharing. We were able to create a similar situation of inequality to test the effect of social-contextual factors on young children’s food sharing behavior (direct “very close” replication; LeBel et al., 2018). An overview and comparison of the results by Birch and Billman and those of the current study can be found in Table 1. We discuss some notable similarities and differences.

Birch and Billman (1986) were not able to support their hypothesis that children would more readily share non-preferred food compared to preferred food. In the current study, however, we did find that children were more likely to share disliked food compared to more favored food. This is in line with our hypothesis and previous research (Blake and Rand, 2010; Shaw and Olson, 2013). Interestingly, after excluding dumping from sharing behavior the difference between sharing preferred and non-preferred food was no longer present (see Supplementary information). Thus, a substantial portion of the non-preferred food that was given to the recipient turned out to be part of dumping unappetizing food that the recipient not wanted, instead of being an act of prosociality (i.e., to benefit others).

As expected, like Birch and Billman (1986) we found a relationship*sex interaction for non-preferred food, but in the opposite direction: Girls gave more non-preferred food to acquaintances than to friends, whereas boys actually shared more non-preferred food with friends than with acquaintances. A similar interaction emerged for spontaneous sharing, indicating that girls also spontaneously shared more non-preferred food with acquaintances than with friends. So, girls in our study seem to want to spare their friends from unattractive food, whereas boys were less considerate with friends (note that Birch and Billman did not find differences in boys’ sharing with friends and acquaintances). This is in line with differences in interaction patterns between male and female dyads described in the Introduction. Moreover, boys are more likely than girls to endorse status-oriented or agentic goals within relationships (Rose and Rudolph, 2006). A certain amount of competition seems to be normative in boys’ friendships but not in girls’ (Schneider et al., 2005), and it has been suggested that the greater male attention to mastery and status is particularly evident during interactions with friends compared to those with nonfriends (Hartup et al., 1993). Regarding our study’s findings, it is possible that boys felt more comfortable to assert their power (by giving undesired objects) within friendship dyads as compared to acquaintances. Overall, our results thus better fit with Birch and Billman’s reasoning and the current literature than their (own) results.

Although Birch and Billman (1986) found no relationship*sex interaction for sharing preferred food, they did find that children shared more preferred food with friends than with acquaintances. However, contrary to our expectations, these findings were not replicated in our study. The literature is inconsistent at this point. In line with our findings, some studies found that children shared preferred food with others irrespective of whether the recipient was a friend or not (Berndt, 1981; Rao and Stewart, 1999; Song, 2019), whereas several other studies concluded that the quality of social relationships does affect preschoolers sharing decisions. These studies compared sharing with friends vs. non-friends, disliked peers, strangers, or out-group members (e.g., Fehr et al., 2008; Moore, 2009; Paulus, 2016; Sparks et al., 2017; Lenz and Paulus, 2021). An effect of relationship might emerge more clearly when the social distance between potential sharer and recipient is greater (Komter, 2010). Nevertheless, we did find an effect of relationship on the type of sharing. In line with the findings by Birch and Billman, we found that friends were more active elicitors than acquaintances. Our study extended these findings by showing that this also applied to passive sharing (i.e., friends were allowed to take away food more often than acquaintances).

Birch and Billman (1986) concluded that successful experiences as a recipient (i.e., been shared with) facilitated sharing. In the present study, the only effect of previous experience was found for passive sharing (i.e., children with no previous experience were more likely to allow food to be taken from their plates), which probably has to do with familiarity with the setting. We did not find an effect of the *quality* (been shared with or not) of the experience. Compared to Birch and Billman, relatively many children in our study had a positive previous experience as a recipient and those who had not often still shared food. Since prosocial behavior, such as sharing, was highly stimulated at both the preschool and elementary school included in our study, this might have contributed to our observation that children behaved prosocially regardless of the quality of their previous experiences. Notably, there was also more sharing (particularly spontaneous) in this study than in the original one. The fact that Dutch preschoolers showed more sharing than American preschoolers 35 years ago, may also point to the role of environmental influences. For example, societal and educational changes have resulted in a shift in educational goals over the last decades. Instead of the traditional focus on cognitive development, social–emotional skills are now also highly valued and often included in a school’s formal curriculum as important learning goals (Van Daalen and De Regt, 2003; D’Emidio-Caston, 2019).

While Birch and Billman (1986) did not find any effects of age, our findings showed that older children (>50 months) shared more preferred food with others compared to younger children (≤50 months). Moreover, passive sharing was more frequent among older children than younger children. These findings are in line with other studies suggesting that sharing behavior increases with age (Fehr et al., 2008; Blake and Rand, 2010; Malti et al., 2016). Whereas young children tend to behave selfishly, older children increasingly prefer resource allocations that removes inequality (Fehr et al., 2008). Moreover, older children may be more capable to consider the wishes and needs of their peers (i.e., perspective-taking) and give normative/moral considerations more weight (Smith et al., 2013). Combined with a stronger inequality aversion, this may result in higher levels of sharing behavior compared to the younger children in our study. Note that the division in age groups corresponds with the age at which children in the Netherlands enter elementary school (Kindergarten), which is at 4 years.

### Strengths, limitations, and future directions

Overall, we managed to perform a direct replication of Birch and Billman’s (1986) classical study using a larger sample size (i.e., increasing the power of our findings). Only direct replications with methodology sufficiently similar to that of the original study, can provide the sort of strict falsification attempt that is needed (LeBel et al., 2018). Nevertheless, some limitations should be noted. First, to facilitate direct comparisons of results (LeBel et al., 2018), the nature of our sample was similar to that of Birch and Billman. However, this reduced the representativeness with respect to the population of Dutch children. That is, our sample consisted of mainly White children from a middle- to upper-class neighborhood who attended the same school site. Sharing behavior should also be examined in more diverse samples (e.g., Schäfer et al., 2015; Dirks et al., 2018). Second, only a few studies have looked at the value of resources. The relationship*sex interaction was only found for sharing non-preferred food, showing the importance of including resources that differ in value. However, some sharing of non-preferred food can be considered dumping with no costs for the sharer (see Supplementary information). We therefore recommend future researchers to focus on the sharing of objects that are desired to a lesser or greater degree. Finally, future work could also benefit from focusing on the strategies children use to acquire food as recipient when confronted with a situation in which objects are unequally distributed (Vermande and Sterck, 2020). More insight into the strategies that are more or less successful in obtaining resources may contribute to a deeper understanding of young children’s sharing behavior.

### Conclusion

Our study showed effects of food preference (i.e., children shared non-preferred food more easily than preferred food), age (i.e., older children shared more food than younger children), and type of relationship (i.e., friends made more active attempts to get food than acquaintances but did not receive more). The effect of type of relationship was qualified by sex and food preference (i.e., girls gave more non-preferred food to acquaintances, but boys gave more to friends). Children who had a negative experience as a recipient (i.e., not shared with) often still shared food when they were potential sharers themselves. These results showed only a low degree of agreement with the original study of Birch and Billman (1986). This was also true when the dumping of disliked food was disregarded as prosocial sharing, as Birch and Billman advised but did not do themselves. However, we also found support for some unconfirmed hypotheses of Birch and Billman, such as the effect of object value and age on sharing behavior. Overall, our study underscores both the need for replications and for studying the effect of social-contextual factors on young children’s sharing behavior in (semi-)natural settings.

## Data availability statement

The data for the current study are not publicly available, but are available from the corresponding author upon reasonable request.

## Ethics statement

The studies involving human participants were reviewed and approved by the Faculty of Ethics Review Board (FERB) of the Faculty of Social and Behavioural Sciences, Utrecht University, the Netherlands. Written informed consent to participate in this study was provided by the participants’ legal guardian/next of kin.

## Author contributions

MV, EvL, and ES conceptualized the study. EH collected and analyzed the data and wrote the first draft of the manuscript. MV made critical revisions. All authors contributed to the article and approved the submitted version.

## Funding

This work was funded by the Dutch Research Council, grant number 401.18.047 awarded to ES and MV. EvL was funded by the European Union under ERC Starting Grant no. 101042961 – CULT_ORIGINS. Views and opinions expressed are however those of the author(s) only and do not necessarily reflect those of the European Union or the European Research Council Executive Agency. Neither the European Union nor the granting authority can be held responsible for them.

## Conflict of interest

The authors declare that the research was conducted in the absence of any commercial or financial relationships that could be construed as a potential conflict of interest.

## Publisher’s note

All claims expressed in this article are solely those of the authors and do not necessarily represent those of their affiliated organizations, or those of the publisher, the editors and the reviewers. Any product that may be evaluated in this article, or claim that may be made by its manufacturer, is not guaranteed or endorsed by the publisher.

## Supplementary material

The Supplementary material for this article can be found online at: https://www.frontiersin.org/articles/10.3389/fpsyg.2023.1130632/full#supplementary-material

## Footnotes

^1^Birch and Billman (1986) suggested that the lack of a main effect of food preference could be due to not taking "dumping" into account. Therefore, we did not only analyze the data using Birch and Billman’s original coding system, but we also analyzed the data adopting a narrower definition of sharing in which dumping behavior was not considered as sharing (i.e., excluding food that did not involve either self-sacrifice by the sharer or benefit to the other). See Supplementary information for details.

^2^In Birch and Billman’s (1986) Table, the number of sharing incidents seems to be accidentally mistaken for the number of pieces of food and vice versa, since the distinction between successful and unsuccessful attempts can only be made for sharing incidents.
