# Supplementary material for: Food sharing with friends and acquaintances: A study in preschool boys and girls
Source: Front Psychol. 2023 Mar 9;14:1130632. doi: 10.3389/fpsyg.2023.1130632 (PMC10034191; doi:10.3389/fpsyg.2023.1130632)
Supplement: Supplementary file 1 [file Table_1.DOCX]

**Appendix A. Results for analyses with adjusted coding**

**Method**

Birch and Billman (1986) explained part of their unexpected results by the fact that they did not take "dumping" into account. Therefore, we did not only analyze the data using Birch and Billman’s original coding system, but we also analyzed the data adopting a narrower definition of prosocial sharing in which dumping behavior was not considered as sharing (i.e., excluding food that did not involve either self-sacrifice by the sharer or benefit to the other). In line with the description of dumping in the Discussion of their paper (Birch & Billman, 1986, p. 394), dumping was coded (i) when the target child gave away non-preferred food despite protest from the recipient (e.g., comments about not wanting the food, giving the food back) or (ii) when the target child gave away non-preferred food in order to get rid of it (e.g., stating that he/she does not want that particular food, giving so-called reasons why the recipient should eat the non-preferred food; for example *“You first have to eat all the cheese [before getting an M&M], it will make you strong” or “You also have to eat the carrots because there are vitamins in them, that’s good for you”*). To create new variables of sharing behavior, the number of pieces of food that were taken back from the recipient *and* the number of pieces of food that were dumped were subtracted from the total number of pieces of food shared. In a similar vein, incidents that only involved dumping behavior were subtracted from the total number of sharing incidents.

Three coders rated the videotapes. To guarantee independence among ratings, no coder rated a target child twice. Coder reliabilities were computed on 22% of the videotapes (*n* = 40). The mean intraclass correlation coefficient (absolute agreement) for dumping was .94 (range .87-1.00).

**Results**

**Quantity of sharing (frequency and amount)**

The mean number of sharing incidents and mean number of pieces of preferred and non-preferred food shared with friends and acquaintances are presented in Table 1, separately for boys and girls. The two measures (i.e., number of sharing incidents and number of pieces of food shared) of preferred food were strongly positively associated, *r* = .81, *p* < .001. The same pattern was found for the measures of non-preferred food, *r* = .57, *p* < .001. When looking at correlations between preferred and non-preferred food, medium positive associations for sharing incidents, *r* = .28, *p* < .01, and pieces of food, *r* = .37, *p* < .001, were found. Similar patterns of correlations were found in the original study (see Birch & Billman (1986), p. 391).

As expected, the mean number of sharing incidents and the mean number of pieces sharing non-preferred food were significantly lower (*ps* < .001) for the adjusted variables (i.e., corrected for dumping behavior) than for the variables based on the original coding of Birch and Billman (1986). Using the original coding, children gave more non-preferred food to their peers than preferred food, but after taking dumping into account we no longer found such a difference in the amount of preferred and non-preferred food shared with others (*p*-values ranged from .232 to .615). Thus, a substantial portion of the non-preferred food that was given to the recipient turned out to be part of dumping behavior rather than ‘real’ sharing.

Using the adjusted variables, we no longer found a significant multivariate interaction between relationship and sex, *Pillai’s F*(4,83) = 2.33, *p* = .063, *η_p_^2^* = .101. However, the univariate analyses showed that the interaction between relationship and sex for the number of pieces sharing non-preferred food still approached significance, *F*(1,86) = 3.89, *p* = .052, *η_p_^2^* = .043. In line with our findings based on the original coding, boys shared more pieces of non-preferred food with friends than with acquaintances, *t*(47) = 2.12, *p* = .039. Surprisingly, girls made no distinction between friends and acquaintances in sharing non-preferred food when dumping was taken into account, *t*(42) = -0.57, *p* = .572. We still found a main effect of age on the number of pieces of preferred food, *F*(1,86) = 4.75, *p* = .032, *η_p_^2^* = .052, with older children sharing more preferred food with their peers than younger children. The effect of age on the number of incidents sharing non-preferred food was, however, no longer significant (*p* = .099). None of the other effects were significant (*p*-values ranged from .067 to .995).

As explained, the correction for dumping behavior caused some changes in the results, particularly regarding the interaction between relationship and sex. Therefore, we took a closer look at the variables concerning dumping behavior in an attempt to explain the different outcomes between the original and adjusted variables. The mean number of incidents and mean number of pieces of food dumped at friends and acquaintances by boys and girls are shown in Table 2. Dumping of food occurred in approximately a third of the dyads (friends: 37.4%; acquaintances: 33.0%) in our sample. Although Birch and Billman (1986) stated in their discussion that dumping was seen exclusively in male dyads, we observed this type of behavior in both boys and girls. A Repeated Measures MANOVA did not reveal any significant effects (*p*-values ranged from .101 to .995), indicating that similar amounts of food were dumped at friends and acquaintances. To additionally inspect whether the interaction effect found for the original coding (i.e., girls shared more non-preferred food with acquaintances than with friends) could at least partly be explained by the dumping behavior of girls, follow-up paired *t*-tests were performed. Results showed that girls indeed dumped more food at acquaintances than friends, *t*(42) = -2.10, *p* = .042, whereas boys dumped similar amounts of food at both friends and acquaintances, *t*(47) = 0.52, *p* = .603. It should be noted, though, that the (univariate) interaction between relationship and sex was not significant, *F*(1,86) = 2.75, *p* = .101, *η_p_^2^* = .031.

**Type of sharing**

For each sharing mode (spontaneous, elicited, passive), Table 3 shows the mean number of sharing incidents and mean number of pieces of food shared with friends and acquaintances separately for boys and girls. The two measures (i.e., number of sharing incidents, number of pieces of food) of spontaneous sharing were strongly positively associated, *r* = .85, *p* < .001. The same pattern was found for elicited sharing, 41, *p* < .001, and passive sharing, *r* = .73, *p* < .001. Notably, the correlations for our measures were somewhat lower than in the original study.

The adjusted mean number of sharing incidents and mean number of pieces of food shared spontaneously (i.e., corrected for dumping behavior) were significantly lower compared to the variables that used the original coding by Birch and Billman (*ps* < .001). A similar pattern was found for the measures for elicited sharing (*ps* < .05). The variables for passive sharing remained the same, which is not surprising given that passive sharing is initiated by the recipient instead of the sharer him/herself.

Using the adjusted variables, we no longer found a significant interaction between relationship and sex for spontaneous sharing (*p* = .073), indicating that the results based on the original coding (i.e., girls spontaneously shared more food with acquaintances than friends) could be attributed to the dumping of non-preferred food. None of the other effects for spontaneous sharing were significant (*p*-values ranged from .075 to .996). The analyses for elicited and passive sharing showed the same results as the analyses using the original coding. Although the multivariate effect of relationship on elicited sharing was not significant, *Pillai’s F*(2,85) = 2.83, *p* = .064, *η_p_^2^* = .063, contrasts still revealed that the number of elicited sharing incidents were higher for friends than acquaintances, *F*(1,86) = 4.15, *p* = .045, *η_p_^2^* = .046. The results for passive sharing stayed exactly the same, because passive sharing did not include aspects of dumping behavior.

Inspection of the success rate of the attempts to get food (see Table 4) revealed similar results as with the original coding. Thus, the greater number of elicited and passive sharing incidents with friends could not be explained by differences in the success rates of attempts (63.7% for friends, 64.4% for acquaintances). Interestingly, the success rates appeared to be higher for passive sharing incidents (76.2% for friends, 81.1% for acquaintances) than for elicited sharing incidents (40.2% for friends, 40.1% for acquaintances).

**Conclusion**

We aimed to replicate and extent the findings of Birch and Billman’s (1986) influential work on preschoolers’ sharing. Following the suggestions of these authors in the Discussion of their paper, we also used adjusted coding in which dumping of disliked food was not considered as prosocial sharing (i.e., excluding food that did not involve either self-sacrifice or benefit to the other). As can be seen in Table 5, only the results for sharing non-preferred food changed when controlling for dumping behavior. This suggests that it may be relevant to differentiate between dumping and actual prosocial sharing behavior when interpreting the results regarding disliked food.

**Table 1**

*Means and Standard Deviations for Sharing Incidents and Number of Pieces of Preferred and Non-Preferred Food Shared with Friends and Acquaintances, Separately for Boys and Girls*

|  | Sex | | | | |  | |  | |  | |  | |
| --- | --- | --- | --- | --- | --- | --- | --- | --- | --- | --- | --- | --- | --- |
|  |  | Boy  (*n* = 48) | | Girl  (*n* = 43) | | Total (*n* = 91) | | | |  | |  | |
|  | *M (SD)* | | *M (SD)* | | *M (SD)* | | Range | | *F*(1,86) | | *η_p_^2^* | |  |
| Incidents P |  | |  | |  | |  | | 1.11 | | .013 | |  |
| Friend | 1.56 (1.47) | | 1.70 (1.37) | | 1.63 (1.42) | | 0-6 | |  | |  | |  |
| Acquaintance | 1.17 (1.24) | | 1.47 (1.49) | | 1.31 (1.36) | | 0-5 | |  | |  | |  |
| Incidents NP |  | |  | |  | |  | | 1.20 | | .014 | |  |
| Friend | 1.31 (1.85) | | 1.12 (1.53) | | 1.22 (1.70) | | 0-8 | |  | |  | |  |
| Acquaintance | 0.85 (1.30) | | 1.12 (1.42) | | 0.98 (1.36) | | 0-6 | |  | |  | |  |
| Pieces P |  | |  | |  | |  | | 0.17 | | .002 | |  |
| Friend | 2.93 (2.51) | | 1.93 (1.64) | | 2.46 (2.19) | | 0-8.5^1^ | |  | |  | |  |
| Acquaintance | 2.04 (2.35) | | 2.27 (2.39) | | 2.15 (2.36) | | 0-10 | |  | |  | |  |
| Pieces NP |  | |  | |  | |  | | 1.79 | | .020 | |  |
| Friend | 2.92 (3.81)^a^ | | 1.58 (2.53) | | 2.29 (3.32) | | 0-10 | |  | |  | |  |
| Acquaintance | 1.64 (2.56)^b^ | | 1.87 (2.44) | | 1.76 (2.50) | | 0-10 | |  | |  | |  |

*Note.* P = preferred food, NP = non-preferred food. *F-*value represents the univariate results for the effect of relationship (friend vs. acquaintance). Different superscripts indicate significant differences within columns.

^1^ If children broke a piece of food in half, only half a piece was scored.

**Table 2**

*Means and Standard Deviations for Dumping Behavior of Non-Preferred Food with Friends and Acquaintances, Separately for Boys and Girls*

|  | Sex | |  |  | | |  | |  | |
| --- | --- | --- | --- | --- | --- | --- | --- | --- | --- | --- |
|  | Boy  (*n* = 48) | Girl  (*n* = 43) | Total (*n* = 91) | | | |  | |  | |
|  | *M (SD)* | *M (SD)* | *M (SD)* | | Range | *F*(1,86) | | *η_p_^2^* | |  |
| Incidents |  |  |  | |  | 0.03 | | <.001 | |  |
| Friend | 0.65 (0.98) | 0.63 (1.07) | 0.64 (1.02) | | 0-4 |  | |  | |  |
| Acquaintance | 0.52 (0.99) | 0.72 (1.26) | 0.62 (1.12) | | 0-6 |  | |  | |  |
| Pieces |  |  |  | |  | 1.68 | | .019 | |  |
| Friend | 2.48 (3.86) | 1.06^b^ (2.11) | 1.81 (3.22) | | 0-10 |  | |  | |  |
| Acquaintance | 2.15 (3.80) | 2.36^a^ (3.83) | 2.25 (3.80) | | 0-10 |  | |  | |  |

*Note. F-*value represents the univariate results for the effect of relationship (friend vs. acquaintance). Different superscripts indicate significant differences within columns.

**Table 3**

|  | Sex | |  |  |  | |  | |  |  |
| --- | --- | --- | --- | --- | --- | --- | --- | --- | --- | --- |
|  | Boy  (*n* = 48) | Girl  (*n* = 43) | Total (*n* = 91) | | |  | |  | |  |
|  | *M (SD)* | *M (SD)* | *M (SD)* | Range | | *F*(1,86) | | *η_p_^2^* | |  |
|  | Spontaneous sharing | | | | | | | | | |
| Incidents |  |  |  |  | | 0.15 | | .002 | |  |
| Friend | 0.81 (1.33) | 1.12 (1.56) | 0.96 (1.44) | 0-5 | |  | |  | |  |
| Acquaintance | 0.81 (1.04) | 1.12 (1.42) | 0.96 (1.24) | 0-6 | |  | |  | |  |
| Pieces |  |  |  |  | | 0.50 | | .006 | |  |
| Friend | 1.33 (2.72) | 0.98 (1.67) | 1.16 (2.28) | 0-10 | |  | |  | |  |
| Acquaintance | 0.96 (1.99) | 1.58 (2.45) | 1.25 (2.23) | 0-9.50 | |  | |  | |  |
|  | Elicited sharing | | | | | | | | | |
| Incidents |  |  |  |  | | 4.15^*^ | | .046 | |  |
| Friend | 1.04 (1.80) | 1.26 (1.33) | 1.14 (1.59)^a^ | 0-8 | |  | |  | |  |
| Acquaintance | 0.83 (1.34) | 0.93 (1.18) | 0.88 (1.26)^b^ | 0-7 | |  | |  | |  |
| Pieces |  |  |  |  | | 0.07 | | .001 | |  |
| Friend | 0.78 (1.64) | 0.74 (1.71) | 0.76 (1.66) | 0-10 | |  | |  | |  |
| Acquaintance | 0.85 (2.31) | 0.76 (1.59) | 0.81 (1.99) | 0-11 | |  | |  | |  |
|  | Passive sharing | | | | | | | | | |
| Incidents |  |  |  |  | | 5.04^*^ | | .055 | |  |
| Friend | 2.15 (2.10) | 1.88 (.195) | 2.02 (2.03)^a^ | 0-10 | |  | |  | |  |
| Acquaintance | 1.19 (1.68) | 1.23 (1.54) | 1.21 (1.61)^b^ | 0-6 | |  | |  | |  |
| Pieces |  |  |  |  | | 2.56 | | .029 | |  |
| Friend | 3.50 (4.01) | 1.79 (2.42) | 2.69 (3.44) | 0-13 | |  | |  | |  |
| Acquaintance | 1.90 (2.78) | 1.78 (2.85) | 1.84 (2.80) | 0-10 | |  | |  | |  |

*Means and Standard Deviations for Spontaneous, Elicited, and Passive Sharing with Friends and Acquaintances, Separately for Boys and Girls*

*Note. F-*value represents the univariate results for the effect of relationship (friend vs. acquaintance). Different superscripts indicate significant differences within columns.

* *p* < .05

**Table 4**

*Mean Number of Successful and Unsuccessful Attempts to Acquire Food*

|  | Successful attempts | Unsuccessful attempts |
| --- | --- | --- |
| Elicited sharing |  |  |
| Friend | 0.43 (0.87) | 0.64 (1.06) |
| Acquaintance | 0.36 (0.64) | 0.52 (1.06) |
| Passive sharing |  |  |
| Friend | 1.54 (1.77) | 0.48 (0.98) |
| Acquaintance | 0.99 (1.44) | 0.23 (0.56) |

**Table 5**

*Overview and Comparison of Results by Birch and Billman (1986) and the Current Study*

|  | Birch and Billman (1986) | Current study | |
| --- | --- | --- | --- |
|  |  | Original coding^1^ | Adjusted coding^2^ |
|  | **Quantity of sharing (frequency and amount)** | | |
|  |  |  |  |
| **Preferred (favored) food** | Relationship: Children shared more preferred food with friends than with acquaintances.  Sex: No effect  Age: No effect  Previous experience: No effect | Relationship: No effect  Sex: No effect  Age: Older children shared more preferred food with others than did younger children.  Previous experience: No effect | Relationship: No effect  Sex: No effect  Age: Older children shared more preferred food with others than did younger children.  Previous experience: No effect |
| **Non-preferred (disliked) food** | Relationship*Sex: Girls shared more non-preferred food with friends than with acquaintances. For boys, patterns of sharing did not differ between friends and acquaintances.  Age: No effect  Previous experience: No effect | Relationship*Sex: Girls shared more non-preferred food with acquaintances than with friends, whereas boys gave more non-preferred food to friends than to acquaintances.  Age: Older children more often shared non-preferred food with others than did younger children  Previous experience: No effect | Relationship*Sex: For girls, patterns of sharing did not differ between friends and acquaintances. Boys shared more non-preferred food with friends than with acquaintances.  Age: No effect  Previous experience: No effect |
| **Food preference** | No effect | Children shared more non-preferred food than preferred food with others. | No effect |
|  |  |  |  |
|  | **Type of sharing** | | |
| **Spontaneous** | Relationship: No effect  Sex: No effect  Age: No effect  Previous experience: No effect | Relationship*Sex: Girls shared more (non-preferred) food spontaneously with acquaintances than with friends, but for boys patterns of sharing did not differ between friends and acquaintances.  Age: No effect  Previous experience: No effect | Relationship: No effect  Sex: No effect  Age: No effect  Previous experience: No effect |
| **Elicited** | Relationship: More food was shared more frequently with friends than with acquaintances.  Sex: No effect  Age: No effect  Previous experience: No effect | Relationship: Elicited sharing was more frequent among friends than acquaintances.  Sex: No effect  Age: No effect  Previous experience: No effect | Relationship: Elicited sharing was more frequent among friends than acquaintances.  Sex: No effect  Age: No effect  Previous experience: No effect |
| **Passive** | Relationship: No effect  Sex: No effect  Age: No effect  Previous experience: No effect | Relationship: Passive sharing was more frequent among friends than acquaintances.  Sex: No effect  Age: Passive sharing was more frequent among older children than younger children.  Previous experience: Passive sharing was more frequent among children without previous experience as a recipient. | Relationship: Passive sharing was more frequent among friends than acquaintances.  Sex: No effect  Age: Passive sharing was more frequent among older children than younger children.  Previous experience: Passive sharing was more frequent among children without previous experience as a recipient. |
|  |  |  |  |
|  | **Previous experience** | | |
| **Type of experience** | A successful experience as a recipient facilitated subsequent sharing, whereas unsuccessful experiences did not. | Children who were not shared with were just as likely to share food as children who were shared with. | Children who were not shared with were just as likely to share food as children who were shared with. |

^1^ Coding based on the variables used by Birch and Billman (1986).

^2^ Coding adjusted for dumping behavior (i.e., excluding dumping behavior from the scores for sharing behavior.
